# A hybrid machine learning approach for automated malaria diagnosis from thin blood smear images

**DOI:** 10.1186/s13071-026-07438-6

**Published:** 2026-05-16

**Authors:** Ghufran Alam Siddiqui, Asish Bera, Ankur Lhila, Amitesh Singh Rajput, Nitika Nitika, Praveen K. Bharti, Ashis Das, Tanmaya Mahapatra

**Affiliations:** 1 Department of Computer Science and Information Systems, Birla Institute of Technology and Science, Pilani, Pilani Campus, Vidya Vihar, Pilani, Rajasthan 333031 India; 2 Department of Electrical and Electronics Engineering, Birla Institute of Technology and Science, Pilani, Pilani Campus, Vidya Vihar, Pilani, Rajasthan 333031 India; 3 Rajiv Gandhi National Cyber Law Centre, National Law Institute University, Kerwa Dam Road, Bhopal, Madhya Pradesh 462044 India; 4 National Institute of Malaria Research, Indian Council of Medical Research, Sector 8, Dwarka, New Delhi, Delhi 110077 India; 5 Molecular Parasitology and Systems Biology Laboratory, Department of Biological Sciences, Birla Institute of Technology and Science, Pilani, Pilani Campus, Vidya Vihar, Pilani, Rajasthan 333031 India

**Keywords:** Malaria diagnosis, Hybrid deep learning, Convolutional neural network, Automated diagnosis, Otsu thresholding, Microscopy

## Abstract

**Background:**

Malaria remains a significant global health challenge, requiring diagnostic approaches that are rapid, cost-effective, and accurate. The present study proposes a hybrid deep learning framework for malaria diagnosis using thin blood smear cell images obtained from the National Institute of Malaria Research (NIMR), a leading research institution of the Indian Council of Medical Research (ICMR).

**Methods:**

A total of 15,938 thin blood smear microscopic images were preprocessed to enhance the visibility of cellular structures and suppress background noise. Segmentation was performed using the Otsu thresholding method, which automatically determines the optimal threshold to maximize cell contrast and improve feature extraction. The processed images were then used to train the proposed Hybrid Inception-v3 convolutional neural network (CNN), specifically designed for automated cell classification. To establish the robustness of the proposed CNN, comparative and statistical analyses were performed against conventional diagnostic techniques and other state-of-the-art machine learning models.

**Results:**

The proposed hybrid CNN model achieved a training accuracy of 98.9%, sensitivity of 97.3%, specificity of 99.9%, along with high F_1_ score and area under the curve (AUC) values. These results were obtained using the ICMR-NIMR microscopic image dataset, demonstrating the robustness and generalizability of our approach. The model consistently outperformed other evaluated methods, including state-of-the-art machine learning classifiers, and demonstrated performance comparable to or better than conventional microscopy-based examinations.

**Conclusions:**

The hybrid deep learning framework demonstrated robust performance for malaria diagnosis using microscopic images, suggesting potential to reduce reliance on expert microscopists. These findings highlight its potential to improve diagnostic precision and strengthen malaria control efforts, especially in resource-limited settings.

**Graphical Abstract:**

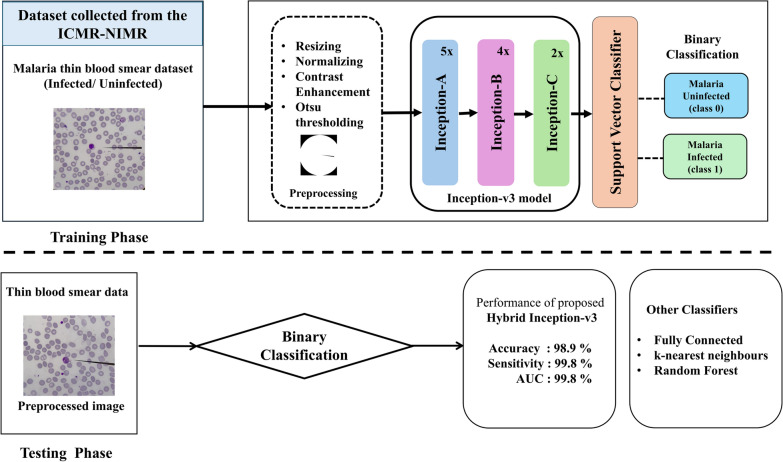

**Supplementary Information:**

The online version contains supplementary material available at 10.1186/s13071-026-07438-6.

## Background

Malaria remains a major global health challenge, with approximately 249 million reported cases and 600,000 deaths annually [1], predominantly affecting low- and middle-income countries in sub-Saharan Africa, which account for nearly 95% of cases. The disease is caused by protozoan parasites of the *Plasmodium* genus and transmitted through female *Anopheles* mosquitoes. Despite significant advances in malaria control, the burden persists due to limited diagnostic access, rising drug resistance, and the reemergence of malaria in previously controlled regions. Eradication efforts in endemic areas are further hindered by inadequate healthcare infrastructure, shortages of diagnostic reagents and trained personnel, insufficient vector control, and weak surveillance systems [2–4].

Accurate and timely diagnosis is critical for patient management and epidemiological surveillance. Although microscopic examination of blood smears remains the gold standard, it is time-consuming, requires skilled personnel, and typically detects parasitemia levels of 50–100 parasites per µL of blood, with analysis taking up to 30 min per slide [5]. Rapid diagnostic tests (RDTs) offer operational advantages but have notable limitations, including false positives due to histidine-rich protein 2 (HRP-2) persistence and genetic variability [6], reduced reliability from HRP2 and histidine-rich protein 3 (HRP3) gene deletions in some regions [7–9], and lower sensitivity of lactate dehydrogenase (LDH)-based assays (75–90%) compared with microscopy, particularly in low-parasitemia or asymptomatic infections [10].

Recent advances in artificial intelligence (AI), particularly deep learning (DL), have significantly improved automated malaria detection from microscopic blood smear images. Convolutional neural network (CNN)-based approaches have demonstrated high performance across multiple studies. For example, Silka et al. [11] achieved 97.1% accuracy using a CNN-based system with a custom imaging setup, while Kumar et al. [12] reported up to 99% precision using VGG-19, ResNet50-V2 and EfficientNet-B3. Narayanan et al. [13] compared fast CNN models, transfer learning approaches and support vector machines (SVMs), achieving 96.7% accuracy (area under the curve [AUC] = 0.9), with DenseNet showing comparable performance. In addition to classification-based models, preprocessing and segmentation strategies have also been explored to enhance detection accuracy. Fatima et al. [14] used bilateral filtering, adaptive thresholding, and morphological operations to isolate parasites, achieving over 91% accuracy with low false positive rates and fast processing times. Similarly, Delgado-Ortet et al. [15] proposed a three-stage framework incorporating red blood cell segmentation, masking, and CNN-based classification, achieving 93.7% accuracy and demonstrating applicability to broader digital pathology tasks.

Further advancements in deep-learning-based malaria detection have focused on improving accuracy, robustness, and real-world applicability. Zhao et al. [16] developed a mobile-based diagnostic system integrating SSD multibox object detection for red blood cell segmentation with a modified VGG-16 model, achieving 96.5% accuracy on National Institutes of Health (NIH) and Broad Institute datasets and enabling offline Android-based screening. Irmak et al. [17] proposed a 20-layer CNN trained on NIH Giemsa-stained images, achieving 95.2% accuracy with class activation map (CAM)-based interpretability. Similarly, Maqsood et al. [18] designed a custom CNN incorporating bilateral filtering and data augmentation, achieving an F_1_ score of 96.8%. Oyewola et al. [19] further enhanced detection performance using a data-augmentation-based CNN integrated with a directed acyclic graph and reinforcement learning and achieved 94.7% accuracy. In addition, Ramos-Briceño et al. [20] developed a ten-layer CNN and advanced preprocessing techniques, achieving 99.5% accuracy for robust species-level classification.

Recent studies have advanced DL-based malaria detection through diverse architectures and practical implementations. Liang et al. [21] presented a 16-layer CNN to classify parasitized and uninfected cells in thin blood smear images, achieving 97.4% accuracy, while Hemachandran et al. [22] reported 99.6% accuracy using a CNN with semantic segmentation and interpretable outputs. Mmileng et al. [23] demonstrated that ConvNeXt-V2-tiny, outperformed Swin Transformer and ResNet with 98.1% accuracy on 606,276 samples. Taye et al. [24] developed a web-based system integrating SVMs and CNNs (VGG-19, Inception-v3 and Xception), achieving 97% accuracy. Similarly, Mujahid et al. [25] showed that EfficientNet-B2 achieved 97.5% accuracy and effectively handled morphologically diverse samples compared with CNN, VGG-16, DenseNet, and Inception-v3. Overall, these studies highlight that performance varies with model architecture, feature extraction strategies and dataset characteristics, emphasizing both the strengths and limitations of existing approaches.

To better understand these variations in performance and methodological differences, a comparative analysis of existing approaches is essential. Table 1 summarizes state-of-the-art machine learning (ML) models applied to the NIH Malaria dataset, highlighting diverse approaches such as fine-tuned CNNs, deep feature extraction methods, and transfer learning with architectures, as well as recent innovations including dilated attention CNNs. Reported accuracies range from 90.8% to 96%, reflecting the impact of architectural design on performance. Recent lightweight and scalable models, such as ConvNeXt-V2 (98.1%) and EfficientNet-v3 (97.5%) have improved accuracy and efficiency, indicating the effectiveness of optimized extraction strategies for biomedical imaging. Motivated by these findings, this study employs pretrained CNN architectures, including Inception-v3, MalariaNet, VGG-16, ResNet-50, and a custom CNN for feature extraction from thin blood smear images, where transfer learning enhances convergence, generalization on imbalanced datasets, and overall classification performance.

**Table 1:** Comparison of different machine learning models on the NIH malaria dataset

Article	ML model	Year	Accuracy (%)
Shekar et al. [35]	Fine-tuned CNN with custom convolutional layers	2019	95.0
Delgado-Ortet et al. [15]	CNN with deep feature extraction	2020	93.7
Sarkar et al. [36]	Fast-CNN (optimized lightweight CNN)	2020	96.0
Saini et al. [37]	VGG-16 (transfer learning approach)	2020	90.8
Alok et al. [38]	Deep CNN with multiple convolutional layers	2020	95.2
Hemachandran et al. [22]	CNN with multilayer feature learning	2021	95.1
Wang et al. [39]	Dilated attention CNN (DACNN)	2022	94.7
Mmileng et al. [23]	ConvNeXt-v2 Tiny (lightweight architecture)	2024	98.1
Mujahid et al. [25]	EfficientNet-v3 (scalable deep CNN)	2024	97.5

CNN, convolutional neural network; DACNN, dilated attention convolutional neural network

Building upon these observations, several DL architectures have been widely adopted for malaria detection due to their feature extraction capabilities. Inception-v3 [26] is optimized for computational efficiency using factorized convolutions and multiscale feature extraction. In this study, pretrained *ImageNet* weights are used with lower layers frozen and higher layers fine-tuned for malaria detection. ResNet-50 [27] employs residual connections to mitigate the vanishing gradient problem, enabling effective training of deep networks while capturing both low-level and high-level features. VGG-16 [28], characterized by its simple architecture with stacked 3 × 3 convolutional filters, has demonstrated strong transferability to medical image analysis tasks. Additionally, MalariaNet [29] is a task-specific CNN designed for automated malaria classification in thin blood smear images, using convolutional and pooling layers to extract discriminative features and accurately distinguish between parasitized and healthy cells.

Despite the remarkable performance of existing DL architectures, several challenges remain prominent. Most approaches rely on end-to-end CNN models with fully connected (FC) layers, which are prone to overfitting when applied to biomedical datasets characterized by class imbalance, limited sample sizes, and high intra-class variability. Furthermore, thin blood smear images often exhibit significant variations in cell morphology, staining intensity, illumination, and background noise, which can adversely affect model generalization and robustness. In such conditions, CNN classifiers may produce unstable decision boundaries, limiting their reliability for clinical applications.

To address these limitations, we propose a hybrid deep learning strategy that combines Inception-v3 for multiscale deep feature extraction with the support vector classifier (SVC) for classification. Inception-v3 effectively captures hierarchical and scale-invariant features from microscopic images, while SVC constructs a margin-maximizing decision boundary that enhances generalization and robustness. By decoupling feature representation from classification, the proposed framework reduces overfitting, improves stability on imbalanced datasets, and achieves more reliable diagnostic performance compared with conventional end-to-end CNN models. The key contributions of this work are summarized as follows:

- Developed a novel hybrid deep learning framework combining Inception-v3 and a SVC for malaria detection. Inception-v3’s parallel convolutional filters extract both fine-grained local and broader global features from thin blood smear cell images, capturing complex morphological patterns, and then these multiscale features are classified by SVC. This hybrid approach has achieved a test accuracy of 98.9% while demonstrating robustness and scalability for automated malaria detection.
- The proposed deep model has been evaluated on a field dataset collected from patient samples at Indian Council of Medical Research (ICMR)–National Institute of Malaria Research (NIMR), demonstrating improved performance over multiple fine-tuned deep learning models and confirming its robustness and effectiveness in real-world malaria detection. Visual explanations further implicate the qualitative effectiveness of the proposed method.

## Methods

### Dataset and data collection

The dataset used in this study was obtained from the ICMR–NIMR^1^ and consists of microscopic thin blood smear images collected from malaria patients. The workflow for blood sample collection, smear preparation, staining, microscopic examination, and image acquisition is illustrated in Additional file 1: Fig. S1. Blood samples were acquired using a finger-prick method and prepared as thin blood smears on glass slides, followed by Giemsa staining to enhance parasite visibility. The stained smears were examined under a binocular light microscope using a 100 × oil immersion objective, and high-resolution images were captured directly through the microscope using a mobile phone camera without additional imaging devices or filters.

The dataset comprises 15,938 blood cell images, including 6717 uninfected and 9221 malaria-infected samples, all annotated at the image level by expert parasitologists. Each image represents an independent microscopic field of view extracted from thin blood smear slides. Although multiple images may originate from the same patient, they correspond to non-overlapping regions and capture distinct cellular information. Due to the absence of patient-level metadata and privacy considerations, this study focuses on image-level classification rather than patient-level diagnosis.

The dataset exhibits natural class imbalance and variability in cell morphology, staining intensity, and background conditions, reflecting real-world diagnostic scenarios. Representative examples of uninfected and malaria-infected blood cell images are provided in Additional file 2: Fig. S2. Prior to model development, images were standardized to a uniform resolution to ensure consistency in preprocessing and training. To evaluate model performance, the dataset was randomly divided into training, validation, and testing subsets using an image-level splitting strategy. The detailed distribution of samples and representative examples are presented in Table 2.

**Table 2:** Dataset distribution for training, validation, and testing images under different split configurations

Split	Train (%)	Train images	Validation (%)	Validation images	Test (%)	Test images
1	80	12,750	10	1594	10	1594
2	70	11,156	20	3188	10	1594
3	60	9562	20	3188	20	3188

Total dataset size = 15,938 images

#### Data preprocessing

Initially, the input images were resized to $200\times 200$ pixels for passing through a CNN. To increase data variability and prevent overfitting, augmentation strategies including random cropping, rotations of 45° and 75°, and Gaussian blurring using a $10\times 10$ kernel were applied. Each image was labeled as 1 (malaria-infected) or 0 (uninfected).

Otsu thresholding [30] was applied to segment cells from the background and reduce noise. This method selects an optimal threshold *T* by minimizing the intra-class variance of foreground and background pixel intensities, defined as:

1 $${\sigma}_{w}^{2}\left(T\right)={\omega}_{0}\left(T\right){\sigma}_{0}^{2}\left(T\right)+{\omega}_{1}\left(T\right){\sigma}_{1}^{2}\left(T\right)$$

where ${\omega}_{0}\left(T\right)$ and ${\omega}_{1}\left(T\right)$ denote the probabilities of the two classes separated by threshold *T*, and ${\sigma}_{0}^{2}\left(T\right)$, ${\sigma}_{1}^{2}\left(T\right)$ are their respective variances. Here, ${\sigma}_{w}^{2}\left(T\right)$ represents the total within-class variance, i.e., the weighted spread of intensities inside the background and foreground regions.

Minimizing ${\sigma}_{w}^{2}\left(T\right)$ ensures that pixels within each class are compact and homogeneous, thereby improving the contrast between cells and background. Since the total variance of image histogram is constant, minimizing ${\sigma}_{w}^{2}\left(T\right)$ is mathematically equivalent to maximizing the between-class variance ${\sigma}_{b}^{2}\left(T\right)$, which is an alternative formulation of Otsu’s method.

**Fig. 1:**
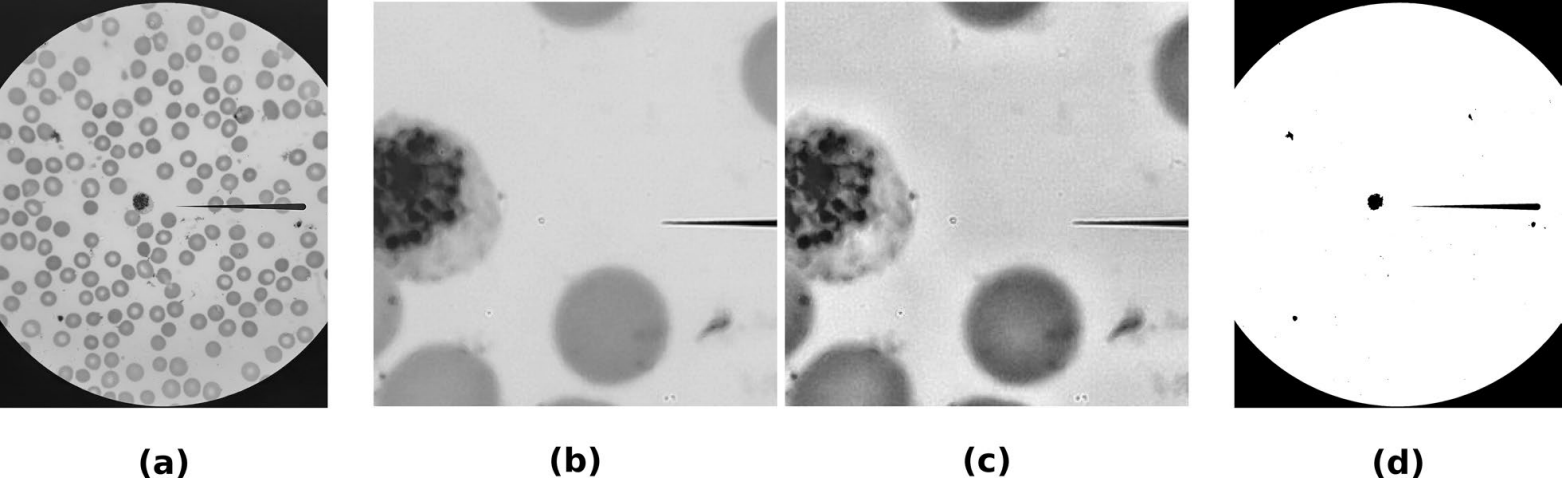
Preprocessing steps including resizing, contrast enhancement, and thresholding applied to thin blood smear images to improve visual quality and highlight diagnostically relevant regions prior to feature learning: **a** original image, **b** resized image, **c** contrast enhancement, and **d** thresholding

Subsequent to image resizing, contrast enhancement was applied to highlight important structural details and improve the distinction between foreground and background regions, thereby enhancing feature visibility. Finally, thresholding was performed to segment regions of interest from the background, reducing noise and simplifying the input for model training. Representative intermediate results of the preprocessing pipeline are illustrated in Fig. 1, including grayscale conversion, cropping to 200 × 200 pixels, contrast enhancement, and Otsu-based segmentation applied to the original image. These steps enhance feature visibility and enable effective separation of foreground structures from the background.

#### Impact of Otsu preprocessing on CNN performance

To explicitly quantify the contribution of Otsu thresholding during preprocessing, we conducted additional experiments comparing multiple CNN-based architectures trained with and without Otsu’s segmentation. Table 3 summarizes the comparative results across multiple experimental runs using accuracy, F_1_ score, and AUC (given in percentage with mean ± standard deviation [SD]). Evident from Table 3, Otsu’s preprocessing consistently improves classification performance across all evaluated architectures, highlighting its effectiveness in separating foreground from background and enabling more robust feature learning in microscopic thin blood smear images.

**Table 3:** Comparison of CNN model performance metrics expressed as mean ± SD (%) with and without Otsu preprocessing

Model	Preprocessing	Accuracy	F_1_ score	AUC
ResNet-50	Without Otsu	71.3 ± 1.1	71.3 ± 1.3	83.2 ± 1.0
	With Otsu	95.5 ± 0.4	94.5 ± 0.4	96.4 ± 0.4
MalariaNet	Without Otsu	83.5 ± 0.9	86.0 ± 0.5	91.5 ± 0.6
	With Otsu	96.2 ± 0.5	95.2 ± 0.5	95.2 ± 0.5
VGG-16	Without Otsu	83.3 ± 0.9	87.1 ± 0.8	92.1 ± 0.6
	With Otsu	97.1 ± 0.3	95.4 ± 0.3	97.1 ± 0.3
Custom CNN	Without Otsu	65.4 ± 1.1	77.0 ± 0.9	72.4 ± 1.6
	With Otsu	96.8 ± 0.4	96.8 ± 0.4	97.3 ± 0.4
Hybrid Inception-v3	Without Otsu	81.9 ± 0.9	85.1 ± 0.8	91.3 ± 0.7
	With Otsu	98.9 ± 0.2	98.8 ± 0.3	98.9 ± 0.1

AUC, area under the curve

### Proposed Hybrid Inception-v3 model

The proposed Hybrid Inception-v3 model excels at learning complex patterns through multiple layers, making it highly effective for medical image classification and suitable for malaria detection from thin blood smear images, as they automatically extract relevant features. The proposed model combines the multiscale feature extraction of Inception-v3 with traditional ML techniques, efficiently capturing diverse features while reducing computational complexity.

#### Phase 1: Baseline model development

We have evaluated multiple DL architectures for malaria detection using thin blood smear cell images, including a custom CNN, VGG-16, MalariaNet, ResNet-50, and a modified Inception-v3 model. All the models were trained to classify images into malaria-infected and uninfected categories, formulating the task as a binary classification problem to learn the mapping

2 $${f}_{\theta }:X\to Y,$$

where an input image set $X\in {R}^{H\times W\times C}$ is mapped to the corresponding class-label $Y\in [\mathrm{0,1}]$, and $\theta$ denotes the trainable parameters. For binary classification, the network outputs a logit $z$, which is converted to a probability using the sigmoid ($\sigma$) activation that maps real values to $\left[\mathrm{0,1}\right]:$

3 $$\widehat{y}=\sigma \left(z\right)=\frac{1}{1+{e}^{-z}}$$

Here, $\widehat{y}$ denotes the predicted probability of the positive class $(y=1$), while $(1-\widehat{y)}$ corresponds to the negative class. Training uses binary cross-entropy loss to measure the discrepancy between predictions and ground-truth labels:

4 $$L\left(\mathrm{y},\widehat{{y}_{n}} \right)=-\frac{1}{N}{\sum}_{n=1}^{N}\left[{y}_{n}\mathrm{log}\left(\widehat{{y}_{n}}\right)+\left(1-{y}_{n}\right)\mathrm{log}\left(1-\widehat{{y}_{n}}\right)\right]$$

where $N$ is the number of training samples, ${y}_{n}\in [\mathrm{0,1}]$ is the ground-truth label of the ${n}^{th}$ sample, and $\widehat{{y}_{n}}$ is the predicted probability of class 1. The sigmoid binary cross-entropy combination provides a stable and computationally efficient optimization framework, with parameters updated using the Adam optimizer for fast and robust convergence.

#### Phase 2: Development of proposed deep learning architecture

A customized baseline CNN was developed to establish initial performance benchmarks in Phase 1. Now, in Phase 2, standard Inception-v3 model [31] composed of Inception-A, Inception-B, and Inception-C modules with parallel convolutional branches, has been adapted for malaria detection to capture multiscale spatial features. To tailor our model for biomedical applications, the actual fully connected layer is replaced with a trainable dense layer followed by dropout to mitigate overfitting on the small dataset. Lower layers are frozen to retain *ImageNet* pretrained weights, while upper layers are fine-tuned to learn domain-specific representations, enabling efficient adaptation with a reduced computational cost.

Each Inception-v3 module applies multiple convolutional filters of varying kernel sizes in parallel, mathematically represented as:

5 $${y}_{i}={f}_{i}\left(x\right)=\mathrm{R}\mathrm{e}\mathrm{L}\mathrm{U}\left({W}_{i}^{\top }x+{b}_{i}\right),\hspace{1em}i\in \{\mathrm{1,2},\mathrm{3,4}\}$$

where ${W}_{i}$ and ${b}_{i}$ represent the learnable kernel weights and biases, and the nonlinear activation function, e.g., rectified linear unit (ReLU). Here, ${y}_{i}$ corresponds to the output feature map from the *i*th branch of the Inception-v3 module, with each branch applying a different operation, such as $1\times 1$, $3\times 3$, or $5\times 5$ convolutions, as well as pooling. The outputs from these parallel branches are then concatenated to form the aggregated feature map:

6 $$y=\mathrm{C}\mathrm{o}\mathrm{n}\mathrm{c}\mathrm{a}\mathrm{t}\left({y}_{1},{y}_{2},{y}_{3},{y}_{4}\right)$$

which integrates fine-grained details and broader contextual information into a unified representation. In this context, ${y}_{1},{y}_{2},{y}_{3},{and y}_{4}$ represent the outputs of the individual branches within the Inception-v3 module. Specifically, ${y}_{1}$ is obtained from the $1\times 1$ convolution branch, ${y}_{2}$ from the $3\times 3$ branch, ${y}_{3}$ from the $5\times 5$ branch (or stacked smaller convolutions in practice), and ${y}_{4}$ from the pooling branch. Each ${y}_{i}$ captures complementary information at different receptive field sizes, and their concatenation enables the model to combine local fine-grained features with broader contextual patterns.

**Fig. 2:**
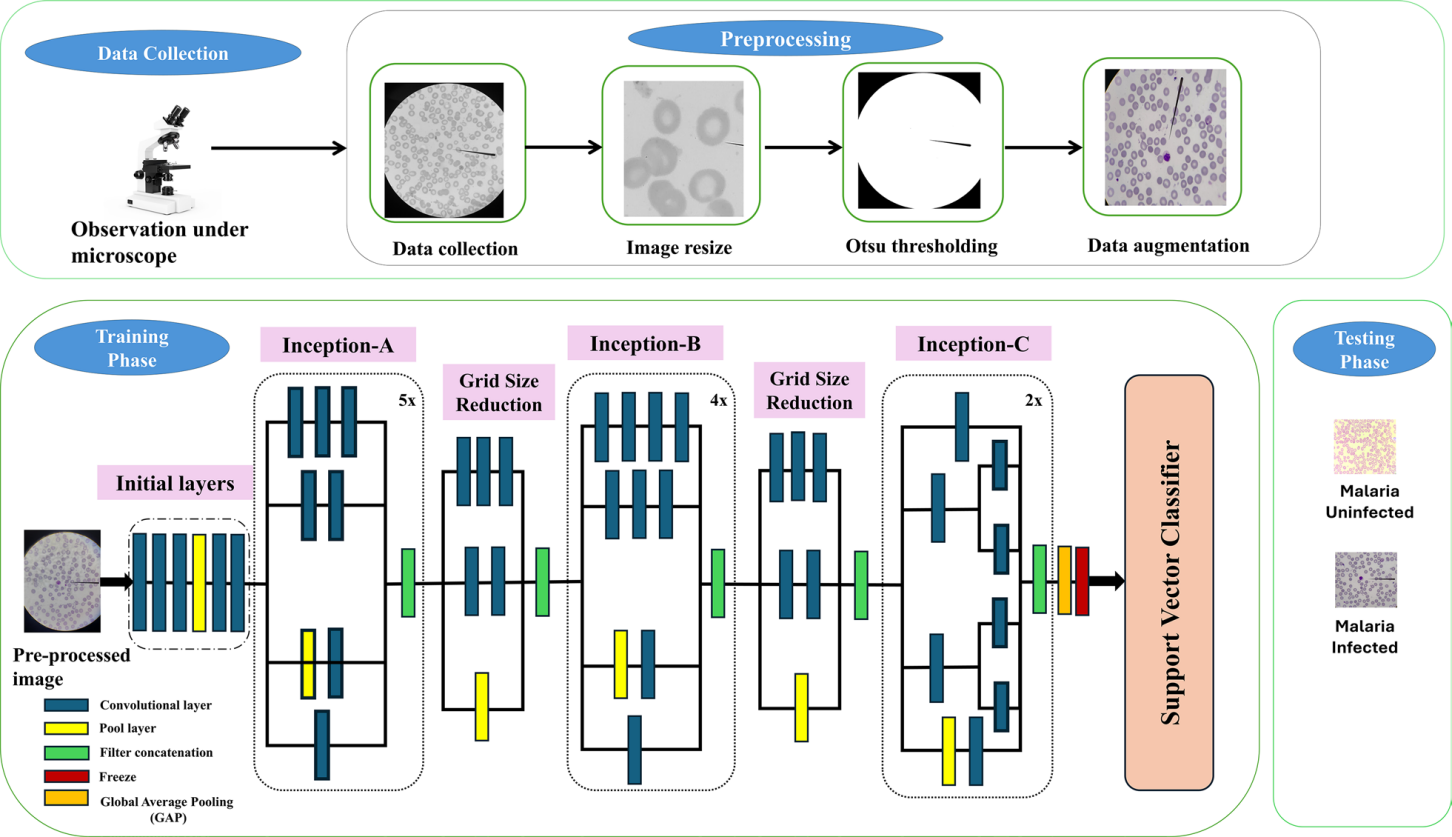
Proposed deep learning architecture for malaria detection using thin blood smear images. The architecture integrates image preprocessing, deep feature learning, and classification to distinguish between malaria-infected and uninfected samples

The architecture of the proposed Hybrid Inception-v3 model is illustrated in Fig. 2. The model emphasizes multiscale feature extraction while balancing accuracy and computational efficiency. Incorporating $1\times 1$ convolutions as bottleneck layers reduces the number of parameters and improves training efficiency. Additionally, parallel convolutional filters enable the network to capture diverse spatial patterns, enhancing generalization across varying image features.

The input is an image of size $200\times 200\times 3$. The initial layers consist of three convolutional operations. The first layer employs 32 filters with a kernel-size of $3\times 3$ and stride of 2, followed by batch normalization and ReLU activation to stabilize learning. This layer is followed by a second convolutional layer with 32 filters and $3\times 3$ kernel-size and a stride of 1. The third layer applies 64 filters, also with $3\times 3$ kernel and a stride of 1. To downsample the data, a max-pooling operation with a $3\times 3$ window size and a stride of 2 is applied, reducing spatial dimension while retaining key features.

After the initial convolutional layers, the feature maps are passed through a sequence of Inception-A blocks. Each Inception-A block has four branches: a $1\times 1$ convolution followed by a $5\times 5$ convolution, a pair of $3\times 3$ convolutions preceded by a $1\times 1$ convolution, and an average pooling layer followed by a $1\times 1$ convolution. The outputs from these branches are concatenated along the channel dimension. Mathematically, this operation is expressed as:

7 $${F}_{out}=\mathrm{C}\mathrm{o}\mathrm{n}\mathrm{c}\mathrm{a}\mathrm{t}\left({F}_{1},{F}_{2},{F}_{3},{F}_{4}\right).$$

Within Inception-A block, intra-concatenation refers to merging the outputs of its four parallel branches; F_1_, F_2_, F_3_, and F_4_. Each branch applies a different transformation (convolutions or pooling), and their concatenation along the channel dimension integrates diverse feature representations at multiple receptive field sizes.

To further reduce spatial dimensions while preserving crucial information, the model incorporates Reduction A blocks. These blocks consist of three branches: a $3\times 3$ convolution with a stride of 2, a $1\times 1$ convolution followed by two $3\times 3$ convolutions (the second with a stride of 2), and a max-pooling operation with a stride of 2. The output from these branches is concatenated. The downsampling operation for a $3\times 3$ convolution in these blocks is given as:

8 $${F}_{\mathrm{r}\mathrm{e}\mathrm{d}\mathrm{u}\mathrm{c}\mathrm{e}\mathrm{d}}=Conv2D\left({F}_{\mathrm{i}\mathrm{n}\mathrm{p}\mathrm{u}\mathrm{t}},W\right)$$

where ${F}_{\mathrm{i}\mathrm{n}\mathrm{p}\mathrm{u}\mathrm{t}}$ is the input feature map, and *W* denotes learned convolutional weights.

Then, the Inception-B block further refines the features. These blocks extend the functionality of Inception-A by introducing $1\times 7$, and $7\times 1$ convolutions, capturing patterns along different spatial axes. The Reduction B block combines $1\times 1$ convolutions, $1\times 7$, $7\times 1$, and $3\times 3$ convolutions along with max-pooling layers for downsampling efficiently. Finally, Inception-C blocks are applied to fine-tune the extracted features. The final feature maps are passed through a global average pooling (GAP) layer, which computes a fixed-size feature vector by aggregating spatial features. The GAP operation is mathematically expressed as:

9 $${x}_{GAP}=\frac{1}{H\times W}{\sum}_{i=1}^{H}{\sum}_{j=1}^{W}{F}_{i,j}$$

where $H$ and $W$ denote the spatial dimensions of the feature map, and ${F}_{i,j}$ represents the feature at position $\left(i,j\right).$ A dropout rate of 0.5 was applied to prevent overfitting, with ReLU activations used in the earlier layers and Leaky-ReLU in later layers to maintain gradient flow in low-activation regions. Additionally, early stopping was employed during training with a patience of five epochs to halt training when validation performance stopped improving, further reducing the risk of overfitting. The Inception-v3 backbone was initialized with *ImageNet* weights, and all layers except the final Inception-v3 block and classification head were frozen:

10 $${W}_{\mathrm{f}\mathrm{r}\mathrm{o}\mathrm{z}\mathrm{e}\mathrm{n}}\left(x\right)=\phi \left(x;{\theta}_{f}\right)$$

where ${\theta}_{f}$ are the fixed pretrained weights, and *x* are the feature maps. This freezing strategy preserved robust low-level visual features while allowing higher-level feature adaptation to the malaria dataset. The proposed Hybrid Inception-v3 architecture contains approximately 18.7 million parameters, compared with approximately 23.8 million parameters in the original Inception-v3, corresponding to a reduction of about 21.4%. This reduction lowers computational complexity while preserving effective feature extraction and the resulting features are classified using an SVC.

#### Phase 3: Fine-tuning and feature extraction

In the second phase of the proposed methodology, pretrained Inception-v3, previously modified with additional convolutional and fully connected layers, is leveraged for transfer learning. Here, in Phase 3, to preserve the learned generic visual features while reducing computational complexity, the initial layers of the Inception-v3 backbone are frozen, ensuring that their weights remain unchanged during the training process. The frozen layers ensure that the network continues to capture primary features—edges, textures, and simple shapes—that are essential for biomedical imaging tasks. The freezing process can be mathematically expressed as:

11 $$W_{frozen} \leftarrow W_{pretrained}, \quad \frac{\partial \mathcal{L}}{\partial W_{frozen}} = 0$$

where ${W}_{\mathrm{f}\mathrm{r}\mathrm{o}\mathrm{z}\mathrm{e}\mathrm{n}}$ denotes the set of frozen layer parameters, ${W}_{\mathrm{p}\mathrm{r}\mathrm{e}\mathrm{t}\mathrm{r}\mathrm{a}\mathrm{i}\mathrm{n}\mathrm{e}\mathrm{d}}$ represents the corresponding pretrained weights, and $L$ is the loss function. The condition $\frac{\partial L}{\partial {W}_{\mathrm{f}\mathrm{r}\mathrm{o}\mathrm{z}\mathrm{e}\mathrm{n}}}=0$

enforces that no gradient updates occur for the frozen layers.

Following this, the output of the modified Inception-v3 network, denoted as ${F}_{out}$ is extracted from the last trainable layer. This output represents a highly informative feature vector that contains discriminative information relevant to the classification task. The extracted feature vector can be represented as:

12 $${F}_{\mathrm{o}\mathrm{u}\mathrm{t}}={\phi}_{\mathrm{m}\mathrm{o}\mathrm{d}\mathrm{i}\mathrm{f}\mathrm{i}\mathrm{e}\mathrm{d}}\left(X;{W}_{\mathrm{t}\mathrm{r}\mathrm{a}\mathrm{i}\mathrm{n}\mathrm{a}\mathrm{b}\mathrm{l}\mathrm{e}},{W}_{\mathrm{f}\mathrm{r}\mathrm{o}\mathrm{z}\mathrm{e}\mathrm{n}}\right)$$

where $X$ denotes the input image, ${\phi}_{\mathrm{m}\mathrm{o}\mathrm{d}\mathrm{i}\mathrm{f}\mathrm{i}\mathrm{e}\mathrm{d}}$ represents the forward propagation through the modified Inception-v3 architecture, ${W}_{\mathrm{t}\mathrm{r}\mathrm{a}\mathrm{i}\mathrm{n}\mathrm{a}\mathrm{b}\mathrm{l}\mathrm{e}}$ are the weights in the unfrozen layers and ${W}_{\mathrm{f}\mathrm{r}\mathrm{o}\mathrm{z}\mathrm{e}\mathrm{n}}$ are the weights in the frozen layers.

By adopting this two-stage process, freezing most of the pretrained layers, and fine-tuning only the newly added and deeper layers, the model benefits from both robust pre-learned feature extraction and domain-specific adaptation. This ensures efficient training, mitigates overfitting, and enhances the capability to handle biomedical image classification tasks, particularly in scenarios with limited labeled data. Table 4 summarizes the key hyperparameters and architectural details of the Inception-v3 and custom CNN models.

**Table 4:** Hyperparameters and architectural details of the Inception-v3 and custom CNN models

Parameter	Inception-v3	Custom CNN
Depth	48	8
Input size	$200\times 200$	$200\times 200$
Optimizer	Adam	Adam
Loss function	Binary cross-entropy	MAE
Batch size	32	32
Learning rate	0.0001	0.007
Momentum	0.9	0.8
Gradient	L2 norm	L2 norm
Weight initializer	Glorot uniform	Glorot uniform

CNN, convolutional neural network; MAE, mean absolute error; L2, ridge regression

Both models employ the Glorot uniform (*Xavier* initialization) weight initializer, which sets the initial weights $W$ according to a uniform distribution:

13 $$W \sim U\left(-\sqrt{\frac{6}{n_{\mathrm{in}} + n_{\mathrm{out}}}},\,\sqrt{\frac{6}{n_{\mathrm{in}} + n_{\mathrm{out}}}}\right)$$

where ${n}_{\mathrm{i}\mathrm{n}}$ and ${n}_{\mathrm{o}\mathrm{u}\mathrm{t}}$ are the number of input and output neurons of the layer, respectively. This initialization ensures that the variance of activations remains stable across layers, preventing vanishing or exploding gradients during training.

By carefully adjusting hyperparameters such as learning rate, batch size, and optimizer settings according to network depth, both Inception-v3 and a custom CNN have achieved efficient training convergence. The deeper Inception-v3 benefits from small learning rates and L2 regularization to manage overfitting, while the shallower custom CNN leverages higher learning rates for faster training. Overall, this configuration allows both models to extract meaningful features from input images while maintaining stable and effective learning dynamics, supporting accurate malaria cell classification.

#### Phase 4: Hybrid classification framework

Phase 4 employs support vector classifier (SVC) for the final classification of thin blood smear cell images. The pooled (GAP) feature vectors obtained from the modified Inception-v3 network are used as input to SVC, which learns an optimal decision boundary between malaria-infected and healthy cells. By maximizing the margin between classes, SVC enhances generalization to unseen data, making it well suited for binary medical image classification tasks [32]. The SVC decision function is given by:

14 $$f\left(x\right)=\mathrm{s}\mathrm{i}\mathrm{g}\mathrm{n}\left({w}^{\top }x+b\right)$$

where $x$ denotes the extracted feature vector, $w$ is the weight vector defining the separating hyperplane, and $b$ is the bias.

The proposed hybrid framework combines the multiscale feature extraction capability of Inception-v3 with the margin-based classification strength of SVC, resulting in high classification accuracy and robustness. This design enables fast and reliable malaria detection from thin blood smear images, making it particularly suitable for low-resource clinical settings by reducing diagnostic time and laboratory workload.

#### SVC hyperparameter and regularization details

The support vector classifier (SVC) was implemented using *scikit-learn* and trained on deep features extracted from the modified Inception-v3 model. A linear kernel was selected on the basis of preliminary experiments. The regularization parameter was set to ($$C=1.0)$$, providing a balance between margin maximization and misclassification tolerance. Since a linear kernel was used, the gamma parameter was not applicable. No class weighting was applied (class weight = none). The input feature vectors corresponded to the global average pooled output of the Inception-v3 backbone. All remaining parameters were kept at default values to ensure reproducibility.

#### Rational for using SVC over fully connected layers

Although fully connected (FC) layers are commonly used as classification heads in transfer learning, their performance can degrade when training data are limited, imbalanced, or high-dimensional. In contrast, SVC optimizes a margin-based objective that promotes better class separation and improved generalization. When applied to deep features extracted by Inception-v3, SVC requires fewer trainable parameters and yields more stable decision boundaries, making it particularly suitable for biomedical image classification tasks where robustness and reliability are critical.

### Evaluation metrics

The proposed model was evaluated using standard performance metrics widely adopted in medical image classification, including accuracy, sensitivity, specificity, and F_1_ score. These metrics are particularly critical for malaria detection, where misclassification can have serious clinical implications. Correct identification of infected cells (true positives) ensures patient safety, while minimizing false positives helps prevent unnecessary treatment.

$$\mathrm{A}\mathrm{c}\mathrm{c}\mathrm{u}\mathrm{r}\mathrm{a}\mathrm{c}\mathrm{y}=\frac{\mathrm{T}\mathrm{P}+\mathrm{T}\mathrm{N}}{\mathrm{T}\mathrm{P}+\mathrm{T}\mathrm{N}+\mathrm{F}\mathrm{P}+\mathrm{F}\mathrm{N}}$$

15 $$\mathrm{S}\mathrm{e}\mathrm{n}\mathrm{s}\mathrm{i}\mathrm{t}\mathrm{i}\mathrm{v}\mathrm{i}\mathrm{t}\mathrm{y}\left(\mathrm{R}\mathrm{e}\mathrm{c}\mathrm{a}\mathrm{l}\mathrm{l}\right)=\frac{\mathrm{T}\mathrm{P}}{\mathrm{T}\mathrm{P}+\mathrm{F}\mathrm{N}}$$

$$\mathrm{S}\mathrm{p}\mathrm{e}\mathrm{c}\mathrm{i}\mathrm{f}\mathrm{i}\mathrm{c}\mathrm{i}\mathrm{t}\mathrm{y}=\frac{\mathrm{T}\mathrm{N}}{\mathrm{T}\mathrm{N}+\mathrm{F}\mathrm{P}}$$

$$F1-\mathrm{s}\mathrm{c}\mathrm{o}\mathrm{r}\mathrm{e}=2\times \frac{\mathrm{P}\mathrm{r}\mathrm{e}\mathrm{c}\mathrm{i}\mathrm{s}\mathrm{i}\mathrm{o}\mathrm{n}\times \mathrm{R}\mathrm{e}\mathrm{c}\mathrm{a}\mathrm{l}\mathrm{l}}{\mathrm{P}\mathrm{r}\mathrm{e}\mathrm{c}\mathrm{i}\mathrm{s}\mathrm{i}\mathrm{o}\mathrm{n}+\mathrm{R}\mathrm{e}\mathrm{c}\mathrm{a}\mathrm{l}\mathrm{l}}$$

Here, the $\mathrm{T}\mathrm{P}$, $\mathrm{T}\mathrm{N}$, $\mathrm{F}\mathrm{P}$, and $\mathrm{F}\mathrm{N}$ denote true positives, true negatives, false positives, and false negatives, respectively. To ensure reliable evaluation under class imbalance, additional complementary metrics were employed. Balanced accuracy mitigates bias toward majority classes by averaging sensitivity and specificity. The Matthews correlation coefficient (MCC) provides a robust single-score assessment by incorporating all elements of the confusion matrix, making it well suited for imbalanced datasets. G-Mean evaluates the balance between correctly identifying malaria-infected and uninfected samples by computing the geometric mean of sensitivity and specificity.

16 $$\mathrm{B}\mathrm{a}\mathrm{l}\mathrm{a}\mathrm{n}\mathrm{c}\mathrm{e}\mathrm{d} \mathrm{A}\mathrm{c}\mathrm{c}\mathrm{u}\mathrm{r}\mathrm{a}\mathrm{c}\mathrm{y}=\frac{\mathrm{S}\mathrm{e}\mathrm{n}\mathrm{s}\mathrm{i}\mathrm{t}\mathrm{i}\mathrm{v}\mathrm{i}\mathrm{t}\mathrm{y}+\mathrm{S}\mathrm{p}\mathrm{e}\mathrm{c}\mathrm{i}\mathrm{f}\mathrm{i}\mathrm{c}\mathrm{i}\mathrm{t}\mathrm{y}}{2}$$

17 $$\mathrm{M}\mathrm{C}\mathrm{C}=\frac{\mathrm{T}\mathrm{P}\cdot \mathrm{T}\mathrm{N}-\mathrm{F}\mathrm{P}\cdot \mathrm{F}\mathrm{N}}{\sqrt{\left(\mathrm{T}\mathrm{P}+\mathrm{F}\mathrm{P}\right)\left(\mathrm{T}\mathrm{P}+\mathrm{F}\mathrm{N}\right)\left(\mathrm{T}\mathrm{N}+\mathrm{F}\mathrm{P}\right)\left(\mathrm{T}\mathrm{N}+\mathrm{F}\mathrm{N}\right)}}$$

18 $$G-\mathrm{M}\mathrm{e}\mathrm{a}\mathrm{n}=\sqrt{\mathrm{S}\mathrm{e}\mathrm{n}\mathrm{s}\mathrm{i}\mathrm{t}\mathrm{i}\mathrm{v}\mathrm{i}\mathrm{t}\mathrm{y}\times \mathrm{S}\mathrm{p}\mathrm{e}\mathrm{c}\mathrm{i}\mathrm{f}\mathrm{i}\mathrm{c}\mathrm{i}\mathrm{t}\mathrm{y}}$$

Although accuracy reflects overall classification correctness, it can be misleading for imbalanced datasets and is therefore insufficient on their own for medical diagnosis. Sensitivity is essential for minimizing missed malaria infections, while specificity ensures correct identification of healthy cells and reduces false alarms. The F_1_ score balances precision and recall, making it particularly valuable when infected samples are less frequent but clinically significant. In addition, imbalance-aware metrics such as balanced accuracy, Matthews correlation coefficient (MCC), and G-Mean provide a more reliable assessment by jointly considering performance across both classes and accounting for class imbalance.

## Results

The main aim of this study was to develop an automated deep learning pipeline for accurately distinguishing malaria-infected and uninfected cells in thin blood smear cell images.

**Fig. 3:**
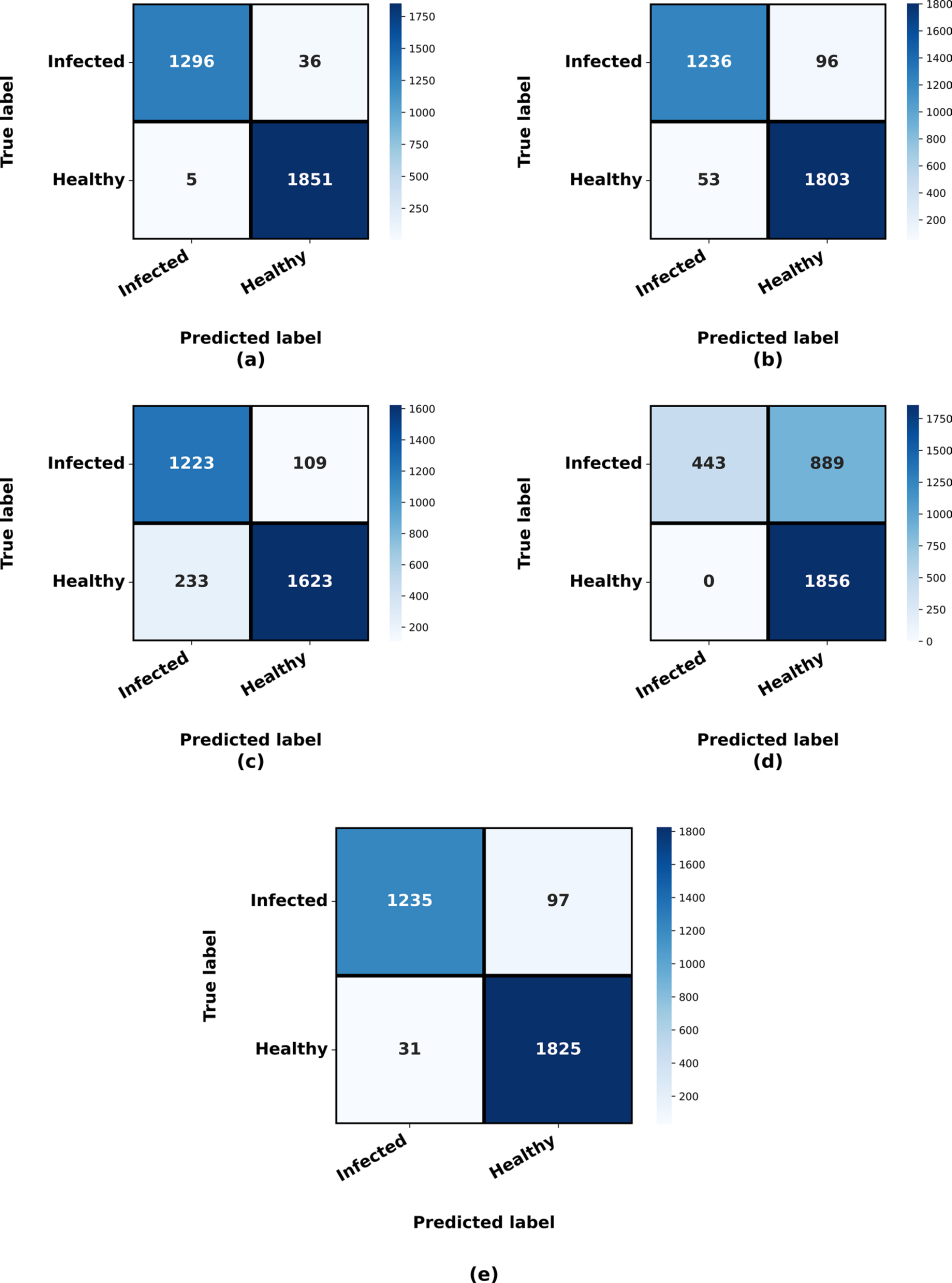
Confusion matrices for malaria cell classification using thin blood smear images. The matrices summarize the distribution of correct and incorrect predictions for malaria-infected and uninfected cells, illustrating the classification performance of different deep learning models. **a** Hybrid Inception-v3, **b** a custom CNN, **c** MalariaNet, **d** ResNet-50, and **e** VGG-16

The confusion matrices of the evaluated models are presented in Fig. 3. The Hybrid Inception-v3 model demonstrates high classification performance, correctly identifying more than 98.0% of both malaria-infected and uninfected cells, with a low incidence of false positive and false negative predictions (Fig. 3a). Similar trends were observed for the other models, with varying degrees of misclassification, as illustrated in Fig. 3b–e. These results indicate the effectiveness of deep features combined with discriminative classifiers for malaria cell classification.

**Fig. 4:**
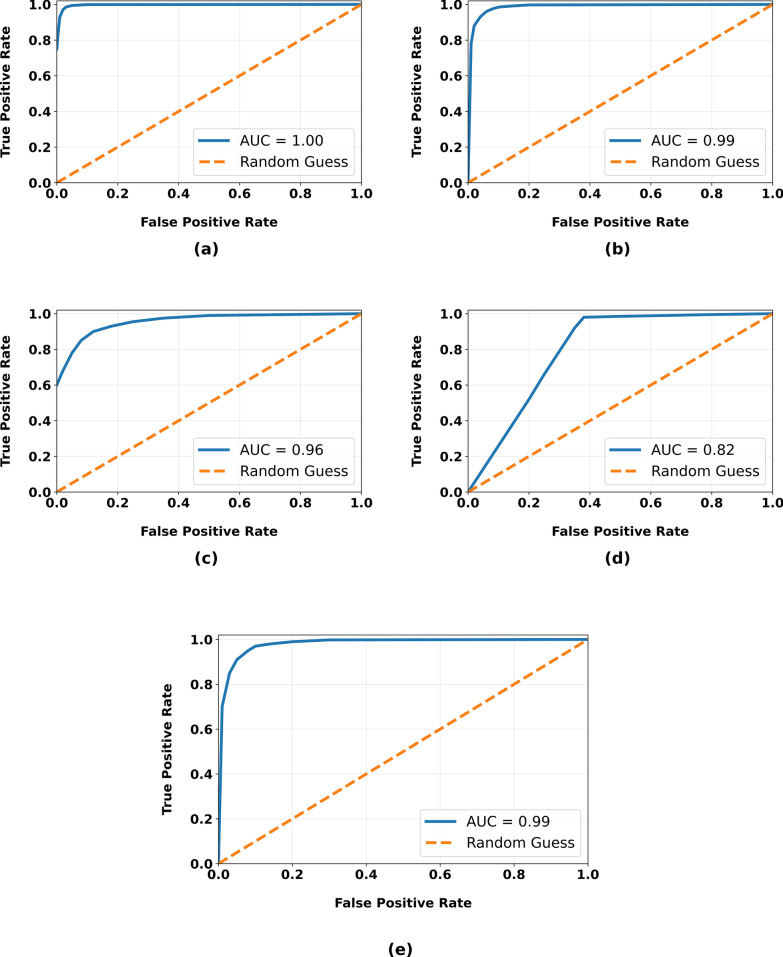
Receiver operating characteristic (ROC) curves and corresponding area under the curve (AUC) values for different classification models. The curves illustrate the trade-off between sensitivity and specificity across different decision thresholds. **a** Hybrid Inception-v3, **b** custom CNN, **c** MalariaNet, **d** ResNet-50, and**e** VGG-16

Table 5 summarizes the key components of the receiver operating characteristic (ROC) curve and AUC in the context of malaria detection. It highlights metrics such as the true positive rate (sensitivity), which measures correct identification of malaria-infected cells, and the false positive rate, which reflects misclassification of healthy cells. These measures are essential for understanding how decision thresholds influence classification performance and result in the receiver operating characteristic (ROC) curve. The ROC curve and corresponding area under the curve (AUC) values of the Hybrid Inception-v3 model and the baseline approaches are presented in Fig. 4. The ROC curves demonstrate the classification performance of all evaluated models across different decision thresholds. Among these, the Hybrid Inception-v3 achieves the highest AUC (Fig. 4a), indicating improved classification performance for thin blood smear cell images, while the remaining models exhibit comparatively lower performance.

**Table 5:** ROC curve elements and AUC interpretation for malaria detection

Term	Description	Interpretation
TPR	Correctly classified parasitized samples	High TPR reduces missed infections
FPR	Uninfected samples classified as parasitized	Low FPR limits false alarms
ROC curve	TPR versus FPR across thresholds	Shows sensitivity–specificity trade-off
AUC	Area under ROC curve (0–1)	Higher values indicate better separation
Threshold	Classification decision boundary	Controls the balance between TPR and FPR

ROC, receiver operating characteristic; AUC, area under the curve; TPR, true positive rate; FPR, false positive rate

**Fig. 5:**
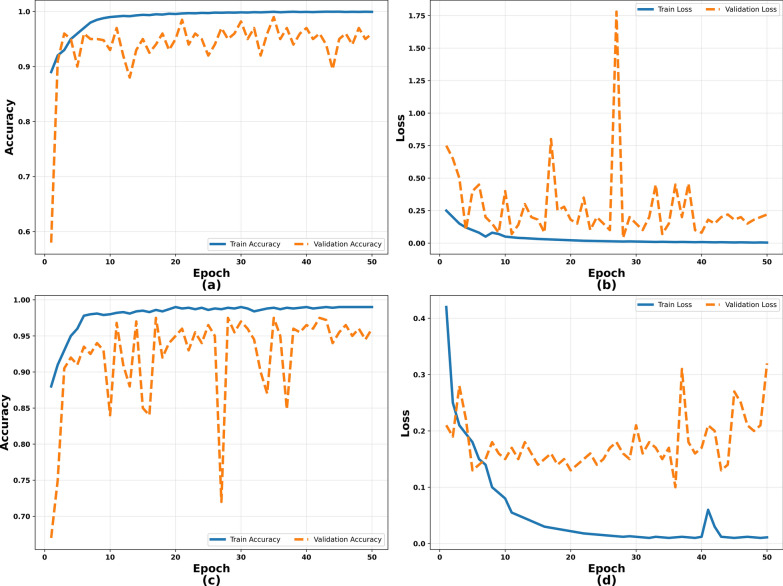
Training accuracy and loss curves for the evaluated models across epochs. The plots illustrate the convergence behavior, training stability, and learning dynamics of the models during optimization. **a** Hybrid Inception-v3 accuracy, **b** Hybrid Inception-v3 loss, **c** custom CNN accuracy, and **d** custom CNN loss

The training and validation accuracy and loss curves of the evaluated models are presented in Fig. 5. The Hybrid Inception-v3 shows smooth convergence with stable training dynamics across epochs (Fig. 5a, b), indicating effective optimization and good generalization performance. In contrast, the custom CNN model converges more rapidly during the initial epoch but exhibits higher variability in validation performance (Fig. 5c, d), suggesting comparatively less stable learning behaviors. Overall, these trends highlight the improved training stability of the Hybrid Inception-v3 architecture.

### Comparative evaluation of custom-built and transfer-learning-based architectures for malaria classification

Table 6 summarizes the performance of five deep learning models: Hybrid Inception-v3, a custom CNN, ResNet-50, VGG-16, and MalariaNet evaluated under three different training–testing-validation split ratios, with all reported values expressed as percentages (%). The Hybrid Inception-v3 model consistently achieved the highest testing accuracy (98.9%) among all models across the different training–testing-validation split ratios (60/20/20, 70/20/10, and 80/10/10). It also demonstrated a higher sensitivity, specificity, and F_1_ score, indicating its robustness in correctly identifying both malaria-infected and uninfected cells. This strong performance highlights the model’s ability to generalize effectively across various data partitions, capturing subtle morphological features in thin blood smear images that other models may miss. The Hybrid Inception-v3 offers a reliable framework for automated malaria diagnosis, potentially reducing diagnostic errors and supporting timely clinical decision-making.

**Table 6:** Performance comparison of deep learning models under different training, testing, and validation splits

Split	Model	Train accuracy	Test accuracy	Sensitivity	Specificity	F_1_ score	AUC
60/20/20	Custom CNN	98.9	95.3	92.8	97.1	94.3	94.9
	ResNet-50	96.5	93.4	83.4	99.4	90.4	94.6
	VGG-16	98.2	95.5	92.7	98.3	95.0	97.0
	MalariaNet	97.5	89.2	91.8	87.4	87.7	89.6
	Hybrid Inception-v3	99.1	98.7	97.3	99.8	98.5	98.7
70/20/10	Custom CNN	99.8	95.3	96.7	96.4	94.6	96.4
	ResNet-50	97.6	95.5	94.9	96.1	93.4	95.7
	VGG-16	98.2	97.0	96.8	97.3	95.0	98.2
	MalariaNet	97.6	96.3	95.9	96.8	94.8	96.7
	Hybrid Inception-v3	99.5	98.3	98.7	98.5	98.7	98.6
80/10/10	Custom CNN	98.1	96.8	96.9	96.6	96.8	97.3
	ResNet-50	96.6	95.5	95.1	96.0	94.5	96.4
	VGG-16	98.3	97.1	96.9	97.4	95.4	97.1
	MalariaNet	97.7	96.4	95.4	96.7	94.3	96.4
	Hybrid Inception-v3	99.9	98.9	99.8	98.7	98.8	99.7

All values expressed as percentages

The results also reveal that data partitioning impacts performance, with the 80/10/10 split generally yielding higher and more stable results, as the larger training set supports better feature learning. While a custom CNN achieved competitive outcomes, particularly in terms of F_1_ score, it lagged the Hybrid Inception-v3. ResNet-50 and VGG-16 showed balanced but moderate performance, whereas MalariaNet, being lightweight, achieved satisfactory accuracy but lower sensitivity and specificity.

#### Performance of a SVC

The SVC achieved an overall classification accuracy of 98.9%, indicating strong predictive performance. It attained a specificity of 98.7%, indicating effective identification of uninfected samples, and a sensitivity of 98.8%, reflecting reliable detection of parasitized cases. The high F_1_ score of 98.8% further confirms a balanced trade-off between precision and recall, underscoring the robustness of the SVC for malaria diagnosis using microscopic thin blood smear cell images.

A comparative analysis of MalariaNet, a custom CNN, Hybrid Inception-v3, ResNet-50, and VGG-16 was conducted to evaluate individual model contributions (Table 6). Among the CNN-based architectures, the Hybrid Inception-v3 achieved the highest accuracy. Although all models demonstrated strong classification performance, variations in sensitivity and specificity indicate that they capture complementary discriminative features from thin blood smear images, supporting the use of hybrid or ensemble strategies to further improve diagnostic accuracy.

#### Comparative evaluation of Inception-v3-based classifiers

We performed a comparative evaluation of multiple Inception-v3-based classification strategies. Specifically, we compared: (i) standard Inception-v3 fine-tuning with a fully connected (FC) classification head, (ii) Inception-v3 features classified using SVC, and (iii) Inception-v3 features classified using alternative machine learning models, including random Forest (RF) and *k*-nearest neighbors (*k-*NN). Performance was evaluated using accuracy, F_1_ scores, and AUC, reported as mean ± SD across multiple runs.

As shown in Table 7, the SVC-based classification framework consistently outperforms both the conventional FC head and alternative machine learning classifiers. This improvement highlights the advantage of margin-based optimization when applied to high-level CNN features, resulting in more stable and generalizable decision boundaries for malaria detection.

**Table 7:** Performance comparison of Inception-v3-based classifiers

Model	Accuracy	F_1_ score	AUC
Inception-v3 + FC	98.0 ± 0.2	98.3 ± 0.2	99.8 ± 0.0
Inception-v3 + RF	98.2 ± 0.1	98.3 ± 0.1	99.0 ± 0.0
Inception-v3 + kNN	98.3 ± 0.1	98.4 ± 0.1	98.9 ± 0.0
Inception-v3 + SVC	98.9 ± 0.2	99.8 ± 0.3	98.9 ± 0.1

Results are reported as mean ± SD (%). FC, fully connected layer; RF, random Forest; kNN, *k*-nearest neighbors; SVC, support vector classifier

### Five-fold cross-validation analysis

To further assess the robustness and generalization capability of the proposed framework, we conducted a five-fold cross-validation study. In each iteration, four folds were used for training and the remaining fold for validation, ensuring that each subset served as the unknown sample set once. This strategy provides a reliable evaluation of model stability while remaining computationally feasible for deep CNN-based architecture.

**Fig. 6:**
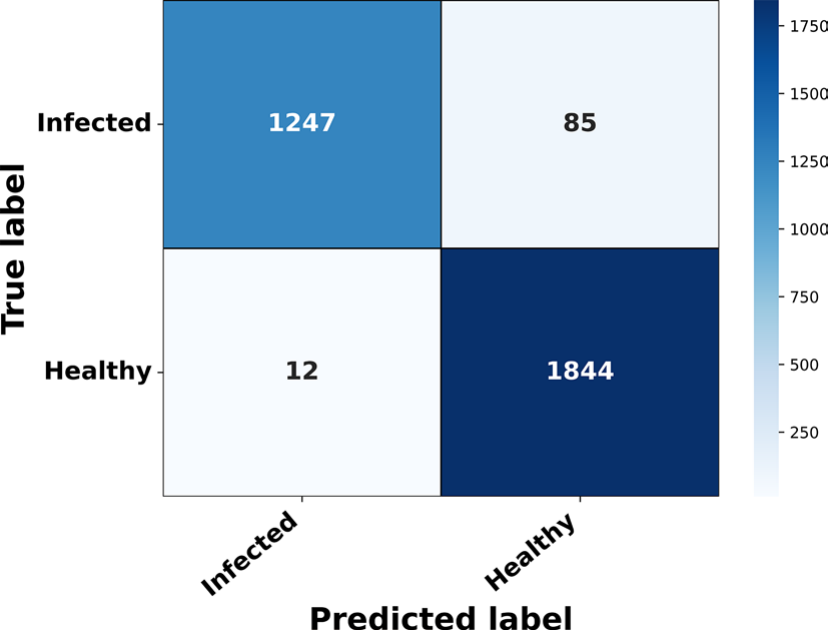
Confusion matrix obtained during five-fold cross-validation. The matrix illustrates the distribution of correctly and incorrectly classified malaria-infected and uninfected cells, providing insight into classification consistency across folds

**Fig. 7:**
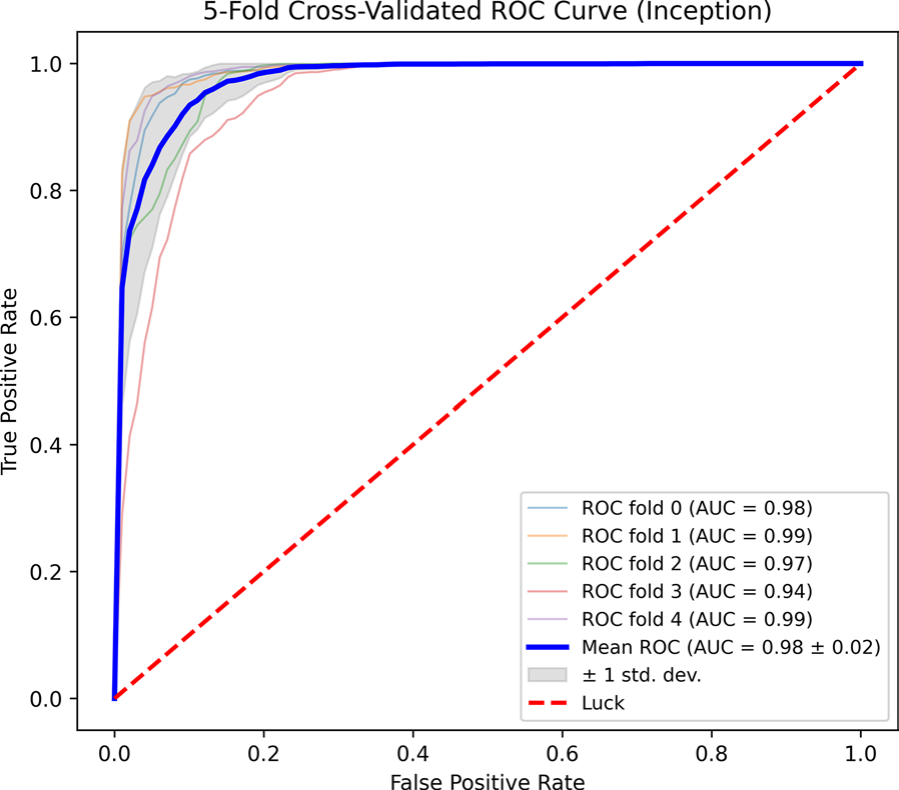
Receiver operating characteristic (ROC) curves across five-fold cross-validation. The figure shows ROC curves for individual folds along with the mean ROC curve and standard deviation band, illustrating the stability and discriminative performance of the model

As shown in Figs. 6 and 7 and Table 8, the proposed model demonstrates consistent performance across all five folds. The confusion matrix reflects a strong balance between correctly classified malaria-infected and healthy samples, while the ROC curves show stable discriminative capability with minimal inter-fold variation. The low standard deviation across metrics confirms the robustness and generalization ability of the proposed framework.

**Table 8:** Per-fold performance of proposed model using five-fold cross-validation (all values expressed as percentages)

Fold	Accuracy	F_1_ score	AUC	MCC	G-Mean
Fold-0	98.2	98.1	98.0	96.5	98.2
Fold-1	98.6	98.6	99.0	97.2	98.6
Fold-2	98.4	98.3	97.0	96.8	98.4
Fold-3	96.8	95.7	94.0	91.8	95.8
Fold-4	98.7	98.7	99.0	97.5	98.7
Mean ± SD	98.2 ± 0.2	97.9 ± 1.1	97.4 ± 2.0	96.0 ± 2.3	98.0 ± 1.1

MCC, Matthews correlation coefficient; G-Mean, geometric mean

### Statistical performance analysis

Tables 9 and 10 present a statistical comparison of the proposed Hybrid Inception-v3 model with baseline architectures using the 80/10/10 split. The results are reported as $mean\pm std$ across multiple runs to capture both performance and stability. For clarity, core classification metrics emphasizing precision, recall (sensitivity), and specificity are reported separately from imbalance-aware measures. The Hybrid Inception-v3 model consistently achieves the highest mean scores with low variance, demonstrating improved accuracy, robustness, and reliable generalization.

**Table 9:** Statistical comparison of classification performance on the 80/10/10 split, with emphasis on accuracy, specificity, F_1_ score, and AUC

Model	Accuracy	Specificity	F_1_ score	AUC
Hybrid Inception-v3	98.9 ± 0.2	98.7 ± 0.3	99.8 ± 0.1	98.9 ± 0.0
Custom CNN	96.8 ± 0.4	96.6 ± 0.8	96.8 ± 0.3	97.3 ± 0.2
VGG-16	97.1 ± 0.2	97.4 ± 0.3	95.4 ± 0.2	97.1 ± 0.0
ResNet-50	95.5 ± 0.4	96.0 ± 0.6	94.5 ± 0.3	96.4 ± 0.1
MalariaNet	96.4 ± 0.5	96.7 ± 1.0	94.3 ± 0.4	96.4 ± 0.2

Results are reported as mean ± SD (%)

**Table 10:** Statistical comparison of imbalance-aware performance metrics on the 80/10/10 split

Model	Balanced accuracy	MCC	G-Mean
Hybrid Inception-v3	99.3 ± 0.2	98.5 ± 0.4	99.3 ± 0.2
Custom CNN	96.9 ± 0.4	94.0 ± 0.8	96.8 ± 0.4
VGG-16	98.7 ± 0.3	97.2 ± 0.6	98.7 ± 0.2
ResNet-50	96.8 ± 0.4	93.3 ± 0.1	96.8 ± 0.4
MalariaNet	94.7 ± 0.6	90.2 ± 1.1	94.7 ± 0.6

Results are reported as mean ± SD (%)

### Explainable artificial intelligence (XAI) for trustworthy malaria detection

Explainable artificial intelligence (XAI) is essential in modern healthcare, where model transparency underpins clinical trust, ethical responsibility, and regulatory acceptance. While traditional models such as decision trees and linear regression are inherently interpretable, CNNs function as black-box models and therefore require additional techniques to explain their decision-making processes.

In this study, two complementary XAI methods, Grad-CAM [33] and LIME [34] were employed to interpret malaria detection results. Grad-CAM is a model-specific approach that generates class-discriminative heatmaps by backpropagating gradients from the predicted class to the final convolutional feature maps, highlighting the most influential image regions. In contrast, LIME is a model-agnostic technique that perturbs the input image and uses a local surrogate model to explain individual predictions, capturing fine-grained features such as cell boundaries, staining patterns, and morphological details.

**Fig. 8:**
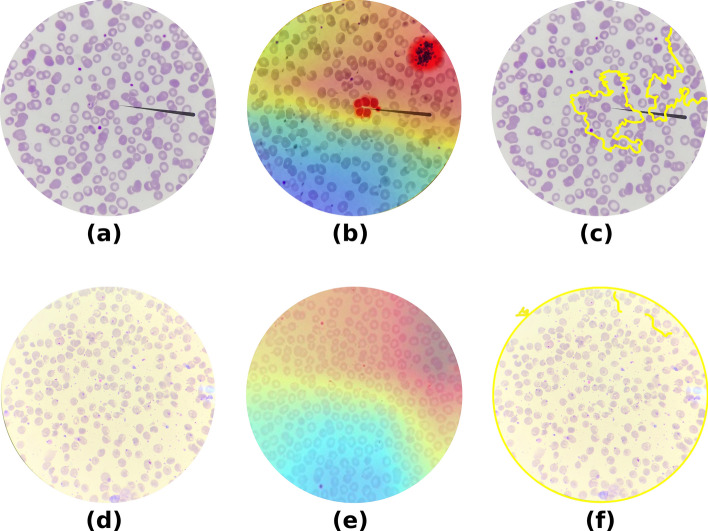
Explainable AI visualization of malaria blood smear images. **a** Original malaria-infected cell image, **b** Grad-CAM visualization, and **c** LIME explanation for the infected sample. **d** Original malaria-uninfected cell image, **e** Grad-CAM visualization, and **f** LIME explanation for the uninfected sample. Grad-CAM and LIME highlight image regions that strongly influence the model’s prediction, supporting interpretability and clinical trust

Figure 8 presents representative examples of the original thin blood smear image alongside Grad-CAM and LIME visualizations. Grad-CAM offers a global interpretation by emphasizing discriminative regions associated with malaria-infected cells, whereas LIME provides localized explanations through super pixel-based regions that most strongly influenced the prediction. Together, these techniques enhance model transparency by confirming that predictions rely on biologically relevant features, supporting reliable clinical malaria diagnosis.

#### Limitations and consistency of XAI explanations

While Grad-CAM and LIME provide complementary insights into the model’s decision-making, their explanations are subject to certain limitations. Grad-CAM offers class-discriminative localization but may produce coarse heatmaps that depend on the selected convolutional layer, whereas LIME generates local, perturbation-based explanations that can vary with superpixel segmentation and sampling. To assess consistency, we observed that, across correctly classified samples both methods consistently highlighted clinically relevant regions, particularly parasite-infected areas and staining patterns, indicating stable explanatory behavior. Nevertheless, variations in explanation granularity may occur, and XAI outputs should be interpreted as a supportive visual aid rather than definitive diagnostic evidence. This combined use of Grad-CAM and LIME enhances transparency while maintaining cautious clinical interpretability.

## Discussion

This study evaluated multiple deep learning architectures for automated malaria detection from thin blood smear images, including ResNet-50, VGG-16, MalariaNet, a custom CNN, and the Hybrid Inception-v3, within a unified experimental framework. While baseline architectures such as VGG-16 and ResNet-50 demonstrated stable performance, and task-specific models such as MalariaNet and custom CNN effectively captured fine-grained morphological features, the proposed hybrid model consistently outperformed them by leveraging multiscale extraction and efficient convolutional operations. This capability enables the model to capture both local cellular details and broader contextual information, which are critical for accurate malaria parasite detection.

The robustness of the proposed framework is further supported by its consistent performance across fixed data splits and *k*-fold cross-validation, with low variability in standard deviation. The incorporation of Otsu-based preprocessing enhances foreground and background separation, facilitating improved feature learning. Analysis of the confusion matrix indicates that predictions are predominantly aligned along the main diagonal, suggesting strong agreement between predicted and true labels and highlighting the model’s ability to effectively distinguish infected cells, even under variations in staining and imaging conditions.

Comparative evaluation on the NIH malaria dataset demonstrates that the proposed hybrid model achieves superior performance, with an accuracy of 98.9%, outperforming other convolutional neural network-based approaches. A detailed comparison with existing state-of-the-art methods is presented in Table 11, where the proposed model achieves superior or comparable accuracy while maintaining a favorable balance between robustness and computational efficiency. Unlike conventional CNN and transfer learning methods, which may suffer from limited generalization or increased computational complexity, the Hybrid Inception-v3 benefits from parallel multiscale feature extraction, enabling improved handling of variations in cell morphology and staining quality commonly observed in real-world clinical data.

**Table 11:** Comparison of malaria detection accuracy on the NIH dataset with previous works

Methods/models	Year	Accuracy (%)
Wang et al. [39]	2022	94.8
Silka et al. [11]	2023	98.6
Mujahid et al. [25]	2024	97.5
Asif et al. [40]	2024	98.5
Shahin et al. [41]	2025	96.4
Proposed Hybrid Inception-v3	2026	98.9

NIH, National Institutes of Health

Furthermore, the proposed approach maintains interpretability while achieving high performance, which is essential for clinical adoption in medical image analysis. Overall, these findings indicate that the hybrid framework is a robust, scalable, and clinically relevant solution for automated malaria diagnosis, with strong potential for deployment in real-world healthcare settings, particularly in resource-limited environments.

## Conclusions

This study presents a hybrid deep learning framework for the detection of malaria parasites in thin blood smear images, aiming to support screening and diagnostic workflows in resource-limited settings. The model, trained and evaluated on carefully annotated datasets including field data from ICMR-NIMR, demonstrated strong classification performance in distinguishing infected and uninfected cells under varying microscopic conditions. Future work will focus on integrating the framework into smartphone-based applications for real-time, point-of-care diagnosis, extending it to patient-level analysis by aggregating predictions across multiple microscopic fields, and enabling multispecies classification (e.g., *Plasmodium vivax* and *Plasmodium falciparum*) along with parasite life cycle stage identification. Additionally, the approach will be expanded to include automated white blood cell analysis for enhanced diagnostic insights, while exploring lightweight deep learning models optimized for mobile and edge devices to support efficient deployment in low-resource environments.

## Supplementary Information

Additional file 1:** Fig. S1.** Overview of the proposed methodology workflow for automated malaria diagnosis using thin blood smear cell images. The pipeline includes data collection, image preprocessing, feature extraction using a deep learning model, and final classification using a machine learning classifier to distinguish between malaria-infected and uninfected cells.

Additional file 2:** Fig. S2.** Representative thin blood smear cell images showing a (a) malaria-infected cell and (b) malaria uninfected cell. The images highlight variations in cell morphology and staining characteristics used for classification.

## Data Availability

The malaria dataset used in this study was provided by the Indian Council of Medical Research–National Institute of Malaria Research (ICMR-NIMR).

## Abbreviations

AI: Artificial intelligence
AUC: Area under the curve
CAM: Class activation mapping
CNN: Convolutional neural network
DACNN: Dilated attention convolutional neural network
DL: Deep learning
FC: Fully connected
FN: False negative
FP: False positive
FPR: False positive rate
GAP: Global average pooling
G-Mean: Geometric mean
HRP-2: Histidine-rich protein 2
HRP3: Histidine-rich protein 3
ICMR-NIMR: Indian Council of Medical Research–National Institute of Malaria Research
kNN: *k*-nearest neighbors
LDH: Lactate dehydrogenase
LIME: Local interpretable model-agnostic explanations
MAP: Maximum a posteriori
MCC: Matthews correlation coefficient
NIH: National Institutes of Health
RDTs: Rapid diagnostic tests
ReLU: Rectified linear unit
RF: Random Forest
ROC: Receiver operating characteristic
SNN: Segmentation neural network
SSD: Single shot detector
SVC: Support vector classifier
SVM: Support vector machine
TN: True negative
TP: True positive
TPR: True positive rate
XAI: Explainable artificial intelligence

## Footnotes

1 https://nimr.icmr.org.in/
