# Supplementary material for: A hybrid machine learning approach for automated malaria diagnosis from thin blood smear images
Source: Parasit Vectors. 2026 May 16;19:283. doi: 10.1186/s13071-026-07438-6 (PMC13348799; doi:10.1186/s13071-026-07438-6)
Supplement: Supplementary file 1 — Additional file 1: Fig. S1. Overview of the proposed methodology workflow for automated malaria diagnosis using thin blood smear cell images. The pipeline includes data collection, image preprocessing, feature extraction using a deep learning model, and final classification using a machine learning classifier to distinguish between malaria-infected and uninfected cells. [file 13071_2026_7438_MOESM1_ESM.pdf]

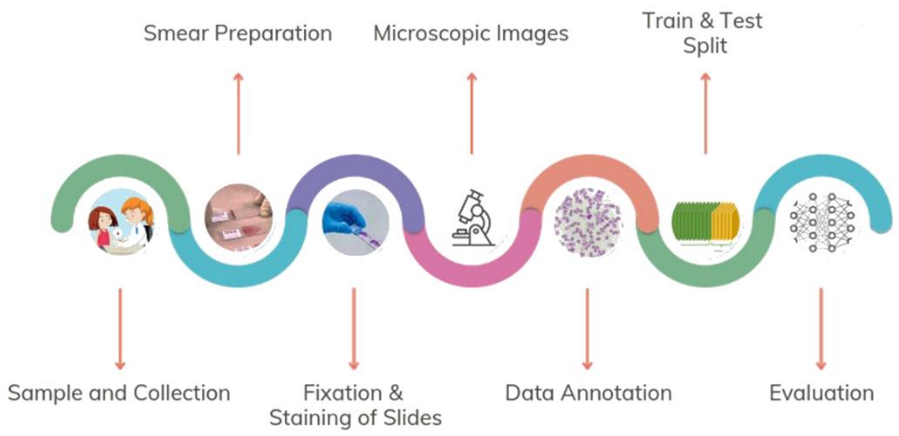

Additional file 1: Fig. S1 Overview of the proposed methodology workflow for automated malaria diagnosis using thin blood smear cell images. The pipeline includes data collection, image pre-processing, feature extraction using a deep learning model, and final classification using a machine learning classifier to distinguish between malaria-infected and uninfected cells.
