# Supplementary material for: A hybrid machine learning approach for automated malaria diagnosis from thin blood smear images
Source: Parasit Vectors. 2026 May 16;19:283. doi: 10.1186/s13071-026-07438-6 (PMC13348799; doi:10.1186/s13071-026-07438-6)
Supplement: Supplementary file 2 — Additional file 2: Fig. S2. Representative thin blood smear cell images showing a (a) malaria-infected cell and (b) malaria uninfected cell. The images highlight variations in cell morphology and staining characteristics used for classification. [file 13071_2026_7438_MOESM2_ESM.pdf]

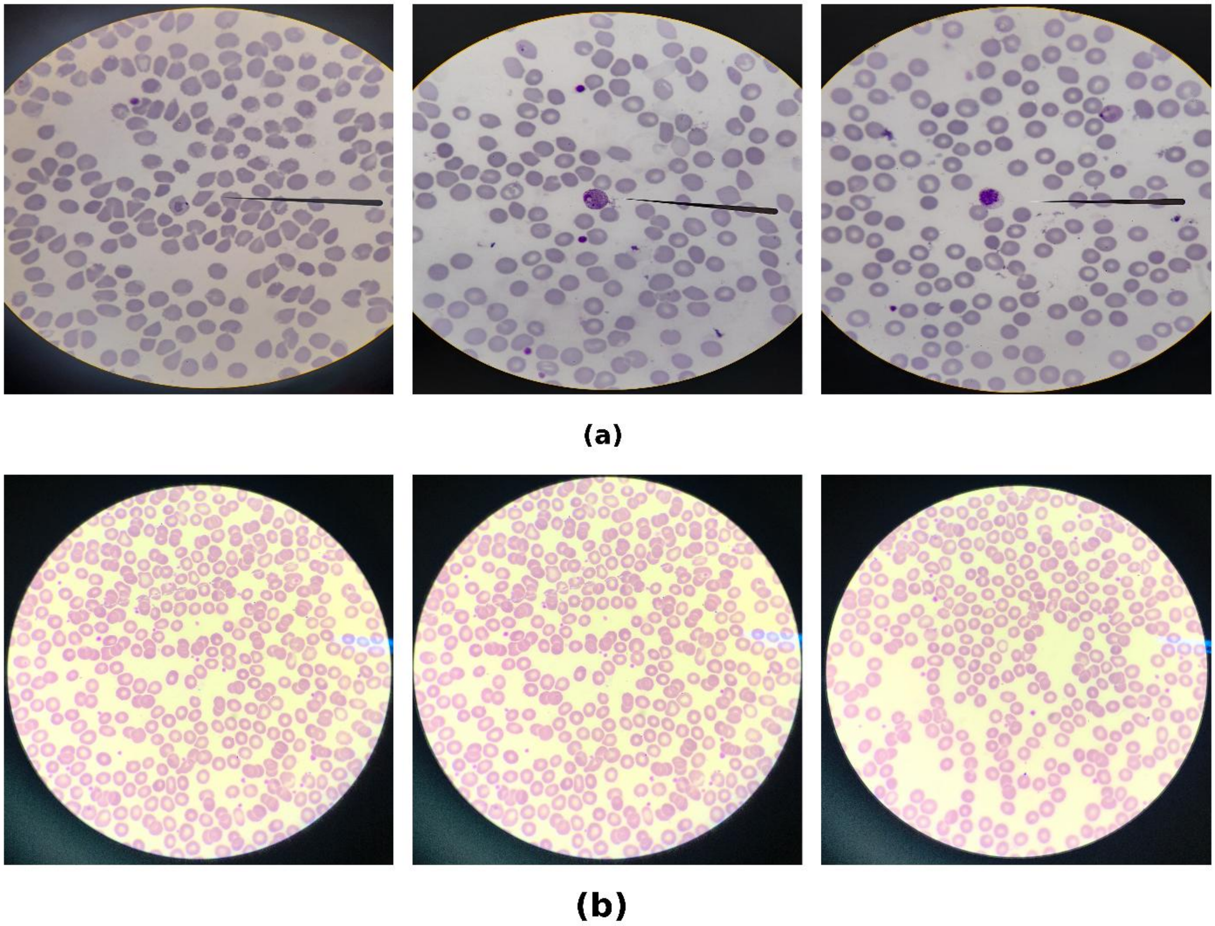

Additional file 2: Fig. S2 Representative thin blood smear cell images showing (a) malaria-infected cell and (b) malaria uninfected cell. The images highlight variations in cell morphology and staining characteristics used for classification.
